# Early evaluation of liver metastasis using spectral CT to predict outcome in patients with colorectal cancer treated with FOLFOXIRI and bevacizumab

**DOI:** 10.1186/s40644-023-00547-w

**Published:** 2023-03-24

**Authors:** Shenglin Li, Long Yuan, Mengying Yue, Yuan Xu, Suwei Liu, Feng Wang, Xiaoqin Liu, Fengyan Wang, Juan Deng, Qiu Sun, Xianwang Liu, Caiqiang Xue, Ting Lu, Wenjuan Zhang, Junlin Zhou

**Affiliations:** 1 Department of Radiology, Lanzhou University Second Hospital, Chengguan District, Cuiyingmen No.82, Lanzhou, 730030 China; 2 Second Clinical School, Lanzhou University, Lanzhou, China; 3 Key Laboratory of Medical Imaging of Gansu Province, Lanzhou, China; 4 Gansu International Scientific and Technological Cooperation Base of Medical Imaging Artificial Intelligence, Lanzhou, China

**Keywords:** Colorectal cancer, Liver metastasis, FOLFOXIRI, Bevacizumab, Efficacy evaluation, Spectral CT

## Abstract

**Purpose:**

Early evaluation of the efficacy of first-line chemotherapy combined with bevacizumab in patients with colorectal cancer liver metastasis (CRLM) remains challenging. This study used 2-month post-chemotherapy spectral computed tomography (CT) to predict the overall survival (OS) and response of CRLM patients with bevacizumab-containing therapy.

**Method:**

This retrospective analysis was performed in 104 patients with pathologically confirmed CRLM between April 2017 and October 2021. Patients were treated with 5-fluorouracil, leucovorin, oxaliplatin or irinotecan with bevacizumab. Portal venous phase spectral CT was performed on the target liver lesion within 2 months of commencing chemotherapy to demonstrate the iodine concentration (IoD) of the target liver lesion. The patients were classified as responders (R +) or non-responders (R −) according to the Response Evaluation Criteria in Solid Tumors (RECIST) v1.1 at 6 months. Multivariate analysis was performed to determine the relationships of the spectral CT parameters, tumor markers, morphology of target lesions with OS and response. The differences in portal venous phase spectral CT parameters between the R + and R − groups were analyzed. Receiver operating characteristic (ROC) curves were used to evaluate the predictive power of spectral CT parameters.

**Results:**

Of the 104 patients (mean age ± standard deviation: 57.73 years ± 12.56; 60 men) evaluated, 28 (26.9%) were classified as R + . Cox multivariate analysis identified the iodine concentration (hazard ratio [HR]: 1.238; 95% confidence interval [95% CI]: 1.089–1.408; *P* < 0.001), baseline tumor longest diameter (BLD) (HR: 1.022; 95% CI: 1.005–1.038, *P* = 0.010), higher baseline CEA (HR: 1.670; 95% CI: 1.016–2.745, *P* = 0.043), K-RAS mutation (HR: 2.027; 95% CI: 1.192–3.449; *P* = 0.009), and metachronous liver metastasis (HR: 1.877; 95% CI: 1.179–2.988; *P* = 0.008) as independent risk factors for patient OS. Logistic multivariate analysis identified the IoD (Odds Ratio [OR]: 2.243; 95% CI: 1.405–4.098; *P* = 0.002) and clinical N stage of the primary tumor (OR: 4.998; 95% CI: 1.210–25.345; *P* = 0.035) as independent predictor of R + . Using IoD cutoff values of 4.75 (100ug/cm^3^) the area under the ROC curve was 0.916, sensitivity and specificity were 80.3% and 96.4%, respectively.

**Conclusions:**

Spectral CT IoD can predict the OS and response of patients with CRLM after 2 months of treatment with bevacizumab-containing therapy.

## Introduction

Colorectal cancer (CRC) is the third most common cancer globally and has a high mortality rate [1]. Approximately 30–50% of patients develop CRC liver metastasis (CRLM) during the course of the disease [2]. Liver resection is the primary curative treatment option for single CRLM, with a 5-year survival of 20–50% [2, 3]. However, most patients with CRLM require first-line chemotherapy including 5-fluorouracil, leucovorin, and oxaliplatin or irinotecan (FOLFOXIRI) [3, 4]. The use of targeted therapies, such as anti-vascular endothelial growth factor (bevacizumab) and anti-epidermal growth factor receptor (cetuximab or panitumumab) monoclonal antibodies, has resulted in increased survival compared with first-line chemotherapy [5–7]. Early assessment of the efficacy of bevacizumab chemotherapy can be helpful in clinical treatment decision making.

Assessment of the response to chemotherapy is based on size criteria, the most common being the Response Evaluation Criteria in Solid Tumors (RECIST) [8]. However, the size-based RECIST v1.1 may not be suitable for early evaluation of the efficacy of bevacizumab for CRLM treatment due to the cytostatic mechanism of bevacizumab [9–11]. In 2009, *Chun *et al*.* [9] proposed morphological assessment criteria for CRLM to assess the response to antiangiogenic therapy in the preoperative setting, which they validated in a nonsurgical patient cohort. Subsequent studies also showed that these criteria were associated with improved long-term prognosis [10, 12, 13]. According to this criterion, based on CRLM changing from heterogeneous masses with ill-defined margins into homogeneous hypoattenuating lesions with sharp borders after bevacizumab treatment is an important basis for response therapy. Besides, m-RECIT criteria is often used to evaluate the efficacy of targeted therapy for liver tumors by assessing the enhancement area in the arterial phase of the lesion. However, the reproducibility and applicability of these criteria derived from naked-eye observations are still limited [11]. In addition, the radiological features of the morphological criteria need to be verified in studies from other centers. As a new imaging analysis method, radiomics has been used to evaluate the effects of treatment on CRLM [11, 14]. However, this method has limited applicability in clinical practice as it is time consuming, labor intensive, and poorly reproducible.

Several quantitative imaging methods, including diffusion-weighted magnetic resonance imaging and F^18^-fluorodeoxyglucose positron emission tomography/computed tomography (PET/CT), have shown good performance in the early response evaluation of chemotherapy for CRLM [15–18]. However, because patients with CRLM require multiple imaging examinations, the high cost of MRI and PET/CT precludes their use in most patients with CRLM. As a new imaging modality, multiparametric spectral CT has been widely used to evaluate liver diseases [19]. Due to its multiparametric imaging characteristics, this method can evaluate tumor cell proliferation [20], microvessel density [21], and fiber structure [22], and it is widely used in clinical practice. In addition, bevacizumab does not lead to reduced size of the lesion in early post-chemotherapy phase but caused decreased iodine concentration. However, to our knowledge, there are no reports of the use of multiparametric spectral CT to assess the efficacy of first-line chemotherapy with bevacizumab-containing therapy in patients with CRLM.

This study was performed to explore the correlation between the overall survival (OS) of patients with CRLM and portal venous phase spectral CT analysis performed within 2 months of commencing first-line chemotherapy with bevacizumab-containing therapy. In addition, we examine whether portal venous phase spectral CT is useful for early identification of CRLM in patients showing a positive response to treatment.

## Material and methods

### Patients

The study was approved by the Ethics committee of Lanzhou University Second Hospital (2022A-298), and the requirement for informed consent was waived due to the retrospective nature of the study.

This retrospective analysis was performed in an initial cohort of 352 patients with pathologically confirmed CRLM between April 2017 and October 2021. These patients received first-line treatment with FOLFOXIRI and bevacizumab. The primary endpoint was OS, defined as the time from inclusion in the study to death or last follow-up. The date of the last follow-up was May 2022. All patients underwent contrast-enhanced CT of the abdomen at baseline and after 6 months of chemotherapy as part of their standard evaluation. Patients underwent at least one spectral CT scan (Time interval between treatment and spectral CT scan: 41.65 ± 6.88, day) between baseline and within 2 months of commencing chemotherapy. These patients were classified based on the RECIST v1.1 at 6 months (6-RECIST v1.1) as responders (R +), defined as a complete response or partial response in the target lesion, or as non-responders (R −), defined as progression, stable disease, or death within 6 months of follow-up.

**Fig. 1:**
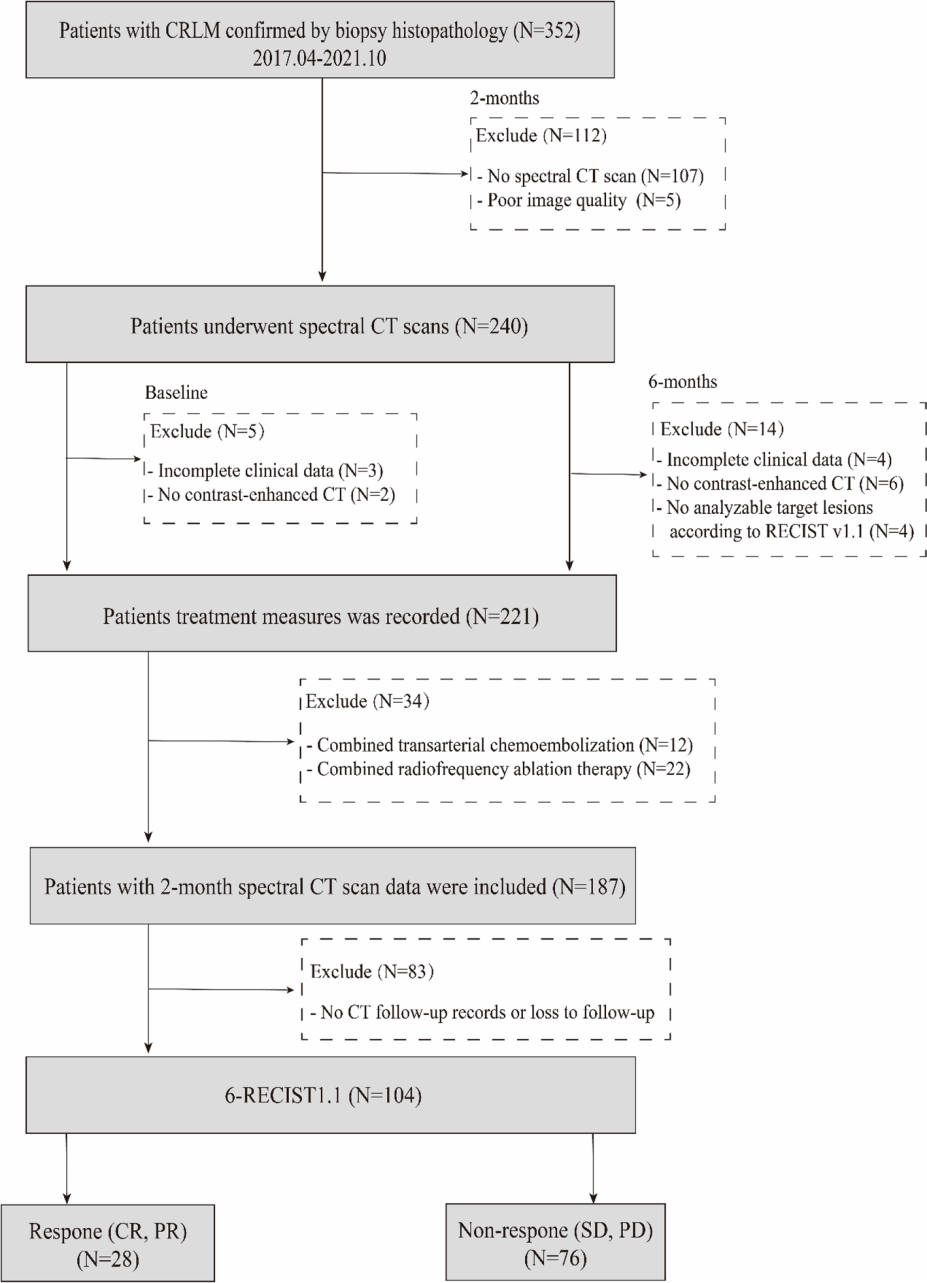
The patient recruitment flowchart

The inclusion criteria were pathologically confirmed CRLM, at least one spectral CT examination within 2 months of starting chemotherapy, abdominal contrast-enhanced CT at baseline and 6 months after chemotherapy, and at least one target lesion > 1 cm at baseline. The exclusion criteria were spectral CT images that could not be analyzed, missing clinical tumor marker results before or during chemotherapy, unknown treatment options, combined transarterial chemoembolization and radiofrequency ablation, and lack of OS data. Finally, 104 patients with CRLM were included in the study. The patient recruitment flowchart is shown in Fig. 1. Radiological (T.L. and L.Y.) Follow-up was performed every 2–4 months with clinical examinations, including blood tests for the tumor markers carcinoembryonic antigen (CEA). Clinicopathological variables were retrieved and manually reviewed via electronic medical records.

### CT imaging

Spectral CT was performed in gemstone spectral imaging mode using the 256-slice Revolution CT scanner or 128-slice Discovery 750HD CT scanner (GE Healthcare, Milwaukee, WI, USA). Baseline and 6-month contrast-enhanced CT was not always performed using the gemstone spectral imaging protocol. The spectral CT scanning parameters were as follows: flat sweep tube voltage, 120 kVp; automatic milliampere-seconds; tube current, 100- 600 mA; collimator width, 0.625 mm; rack speed, 0.6 s/rot; pitch, 0.983:1; and reconstruction layer thickness and layer spacing, 1.25 mm. During enhanced CT, iodixanol (320 mg I/mL) was injected through the anterior cubital vein using a high-pressure syringe at a flow rate of 3.5- 4.0 mL/s and dose of 1.0 mL/kg body weight. The trigger threshold for abdominal aorta monitoring was 100 Hounsfield units (HU). After triggering, the arterial, portal venous, and delay phase scan times were 5, 19, and 90 s, respectively. Moreover, 40% Asir-V iteration was used to reconstruct the portal venous phase spectral-enhanced images horizontally at the end of the scan.

### Image reconstruction processed

40 keV, 50 keV, 60 keV and 70 keV mono energetic CT images were reconstructed. Then, the optimal contrast-to-noise ratio (CNR) was used to determine 70 keV mono energetic CT images as the optimal evaluation data set for target lesions.

### Images and clinical information analysis

Portal venous phase CT is currently the internationally recommended standard imaging technique for follow-up of patients with liver metastasis [11]. Therefore, our analysis of target lesions was based on CT images of the portal venous phase. All abdominal CT scans were reviewed retrospectively and independently by experienced radiologists (T.L., L.Y., Y.X., and M.Y.Y. with 5, 6, 7, and 9 years of experience in the field of abdominal imaging, respectively) blinded to the clinical information of CRLM. Two radiologists (Y.X. and M.Y.Y.) independently analyzed the spectral CT images scanned within 2 months of starting treatment and recorded the morphological and spectral CT parameters of the target lesions. The patients’ tumor markers were documented using the hospital’s Picture Archiving and Communication System (PACS). The energy spectral parameters were averaged. Discrepancies between the radiologists were resolved by consensus review.

Two of the radiologists (T.L. and L.Y.) independently measured the longest diameter (LD) of the target lesion on contrast-enhanced CT at baseline, at spectral scan and at 6 months. In addition, the tumor marker results at baseline, at spectral scan and 6 months were recorded according to the hospital’s PACS. The LD of the target lesion was taken as the average of two radiologists' measurements. One month later, the baseline and 6-month contrast-enhanced CT images from 30 patients were randomly selected, and two radiologists again measured the LD of the target lesions, and the intra- and interobserver reliabilities of the measurements were calculated.

### Qualitative review

According to the classification criteria of *Chun *et al. [9], we classified the morphological criteria of the target lesions as clear or unclear tumor borders and as homogeneous or heterogeneous. We also described the shape of the lesions (round or lobulated) based on the morphology of the target lesions [23]. Morphological classification criteria were assessed on spectral CT images within 2 months of commencing therapy. Liver oligometastasis, defined as the number of metastases between 1 and 5. The LD of the target lesions was measured on spectral CT images according to RECIST v1.1 (2-RECIST v1.1) to allow earlier identification of R + and R − .

### Quantitative review

A region of interest (ROI) with an area of approximately 30 -100 mm^2^ avoiding obvious blood vessels and cystic/necrotic structures was delineated on the target lesion on portal venous phase spectral CT images. Portal venous phase spectral CT analysis was performed using the ROI of the target lesion, including the spectral CT iodine concentration (IoD), spectral curve slope (CS), and normalized IoD (NIoD). The target lesion CS = (CT_40keV_- CT_70keV_) / (70- 40), NIoD = IoD / Aorta IoD (aortic ROI 30 mm^2^). CEA of patients before and after treatment were obtained from electronic medical records. IoD, CEA, CS, and baseline longest diameter (BLD) of the target lesion were assessed as categorical variables using the cut-off values of the survival analysis, in which patients with the greatest reductions (Low) were compared with the remaining patients (High).

According to RECIST v1.1, patients were defined as R + if no target lesion was visible, or a partial response with a > 30% decrease in the target lesion size was observed. R − was defined as disease progression, > 20% increase in target liver lesion size, or stable disease [8]. To facilitate the application of RECIST v1.1, the LD of the target lesion at baseline and 6 months were classified as R + or R − using an online tool (https://www.radiologytutor.com/index.php/ cases/oncol/139-recist). The R + and R − classifications at 6 months, corresponding to the time point used in the literature, were defined as 6-RECIST v1.1 [11].

### Statistical analysis

Continuous variables are expressed as the mean with standard deviation or median with interquartile range (IQR). Categorical variables are expressed as numbers with percentages. Data were tested for normality using Q-Q plots and the Kolmogorov–Smirnov test. Quantitative variables were compared using Student’s t test for normally distributed data or the Mann–Whitney U test for nonnormally distributed data. Qualitative variables were compared using the χ^2^ or Fisher’s exact test. Inter-reader agreement was analyzed using weighted k statistics for qualitative variables and the Spearman correlation coefficient for quantitative variables. A kappa coefficient or correlation coefficient (*r*) > 0.75 was considered to indicate good intra- or interobserver agreement, respectively.

Univariate Cox regression analysis was performed to assess the associations of tumor markers, morphology, and spectral CT parameters with OS as the endpoint. Multicollinearity between independent variables was evaluated through the calculation of the variance inflation factor (VIF). Kaplan–Meier survival curves and the log-rank test were used to test the associations between outcomes with IoD, CS, CEA, and BLD. Factors with *P* < 0.05 in the univariate analysis were included in the multivariate Cox analysis, and the results are expressed as hazard ratios (HR) with 95% confidence intervals (CI). Variables with a VIF ≥ 10 were excluded in order to avoid multicollinearity. The associations of clinical factors, morphology, and spectral CT parameters with respond as the endpoint were assessed by Logistic regression, and the results are expressed as odds ratios (OR) with 95% CI. Receiver operating characteristic (ROC) analyses of the spectral CT parameters significant in the multivariate analysis were performed. The Yuden index was used to calculate cutoff, sensitivity, and specificity values of the parameters with an area under curve (AUC). All statistical analyses were performed using R (version 4.1.2; https://www.r-project.org/), and *P* < 0.05 was taken to indicate statistical significance.

## Results

### Study cohort

The study population consisted of 104 patients with CRLM with a median age of 57.73 (range, 31- 87) years, of whom 42.31% (44/104) were female; 51.00% (53/104) of the primary tumors were rectal cancer. According to the 6-RECIST v1.1, 26.92% (28/104) patients were classified as showing a R + . The total duration of survival follow-up in this study was 48 months. After a median follow-up of 24.0 (range, 9- 42) months, 11.54% (12/104) of the patients were still alive. Baseline data are presented in Table 1.

**Table 1:** Modify the content in the Note of the table

Characteristics		Overall (*N* = 104)	
Age, years		57.73 ± 12.56	
Spectral CT interval, Day		41.65 ± 6.88	
Baseline CEA [median (IQR)]		32.91 (17.00,96.40)	
Sex			
Female		44 (42.31%)	
Liver oligometastasis			
YES		33 (31.73%)	
NO		71 (68.27%)	
Type of liver metastasis			
Synchronous		44 (42.31%)	
Metachronous		60 (57.69%)	
Baseline longest diameter, mm [median (IQR)]		25.74 (20.55,30.18)	
Spectral CT tumor longest diameter, mm [median (IQR)]		26.22 (20.71,32.01)	
6-month tumor longest diameter, mm [median (IQR)]		32.63 (17.51,39.23)	
Sites of metastases			
Liver only		83 (79.81%)	
Liver and extrahepatic	Lung		10 (9.62%)
	Bone		8 (7.69%)
	Others		3 (2.88%)
Systemic therapy scheme			
FOLFIRI and bevacizumab		72 (69.2%)	
FOLFOX and bevacizumab		32 (30.8%)	
Primary tumor			
Rectum		53 (51.0%)	
Colon		51 (49.0%)	
Histologic grade of the primary tumor			
well/moderately differentiated		49 (47.12%)	
Others		55 (52.88%)	
K-RAS			
Wild		49 (47.12%)	
Mutant		55 (52.88%)	
Clinical T stage of the primary tumor			
T2		2 (1.92%)	
T3		43 (41.35%)	
T4		59 (56.73%)	
Clinical N stage of the primary tumor			
N0-1		57 (54.8%)	
N2		47 (45.2%)	
2-RECIST1.1			
R-		98 (94.23%)	
R +		6 (5.77%)	
6-RECIST1.1			
R-		76 (73.08%)	
R +		28 (26.92%)	
Outcomes			
Alive		12 (11.54%)	
Overall survival, month [median (IQR)]		24 (15.00,34.00)	

Note: R* +* Responders; R*−*Non-responders, *2-RECIST v1.1* RECIST v1.1 criteria for spectral CT images within 2 months. 6-RECIST v1.1, RECIST v1.1 criteria for contrast-enhanced CT images at 6 months

### Univariate and multivariate associations of baseline data and spectral CT parameters with OS

**Fig. 2:**
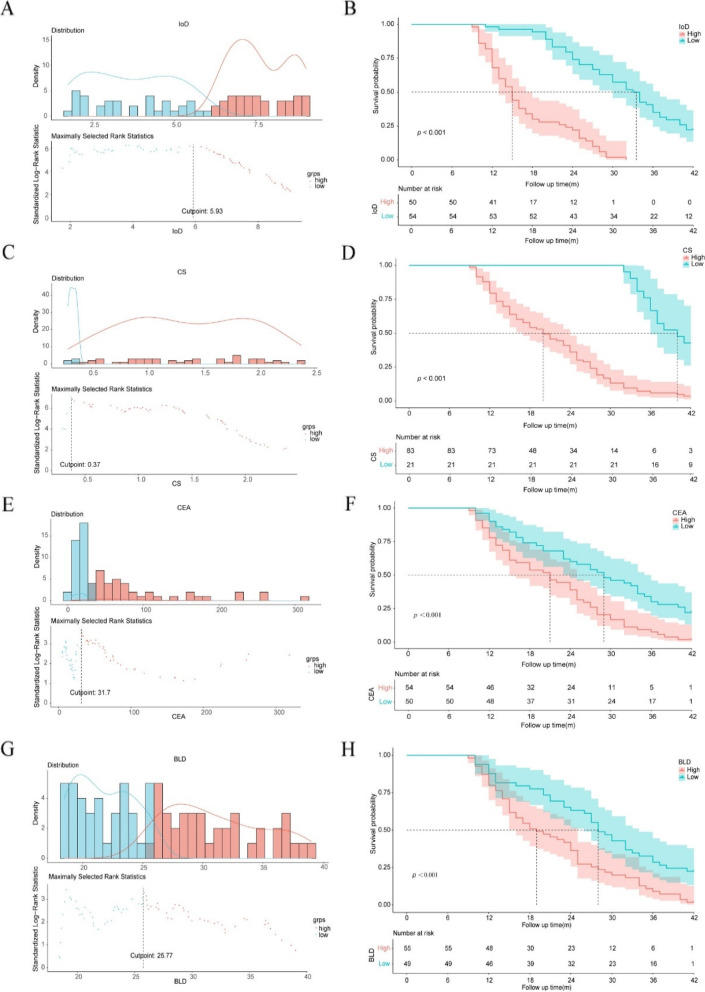
A, B. OS in CRLM patients stratified by IoD = 5.93 (100ug/cm3). C, D. OS in CRLM patients stratified by CS = 0.37. E, F. OS in CRLM patients stratified by CEA = 31.7 (ng/mL). G, H. OS in CRLM patients stratified by BLD = 25.77 mm

Univariate Cox analysis showed that IoD, CS, baseline CEA, BLD, histologic grade of the primary tumor, K-RAS mutation, clinical T stage and N stage of the primary tumor, and metachronous liver metastasis were associated with OS. Patients with CRLM with longer OS had lower IoD [< 5.93 (100ug/cm^3^)] and CS (< 0.37), as well as lower baseline CEA (< 31.7 ng/mL) and BLD (< 25.77 mm) in Fig. 2.

**Fig. 3:**
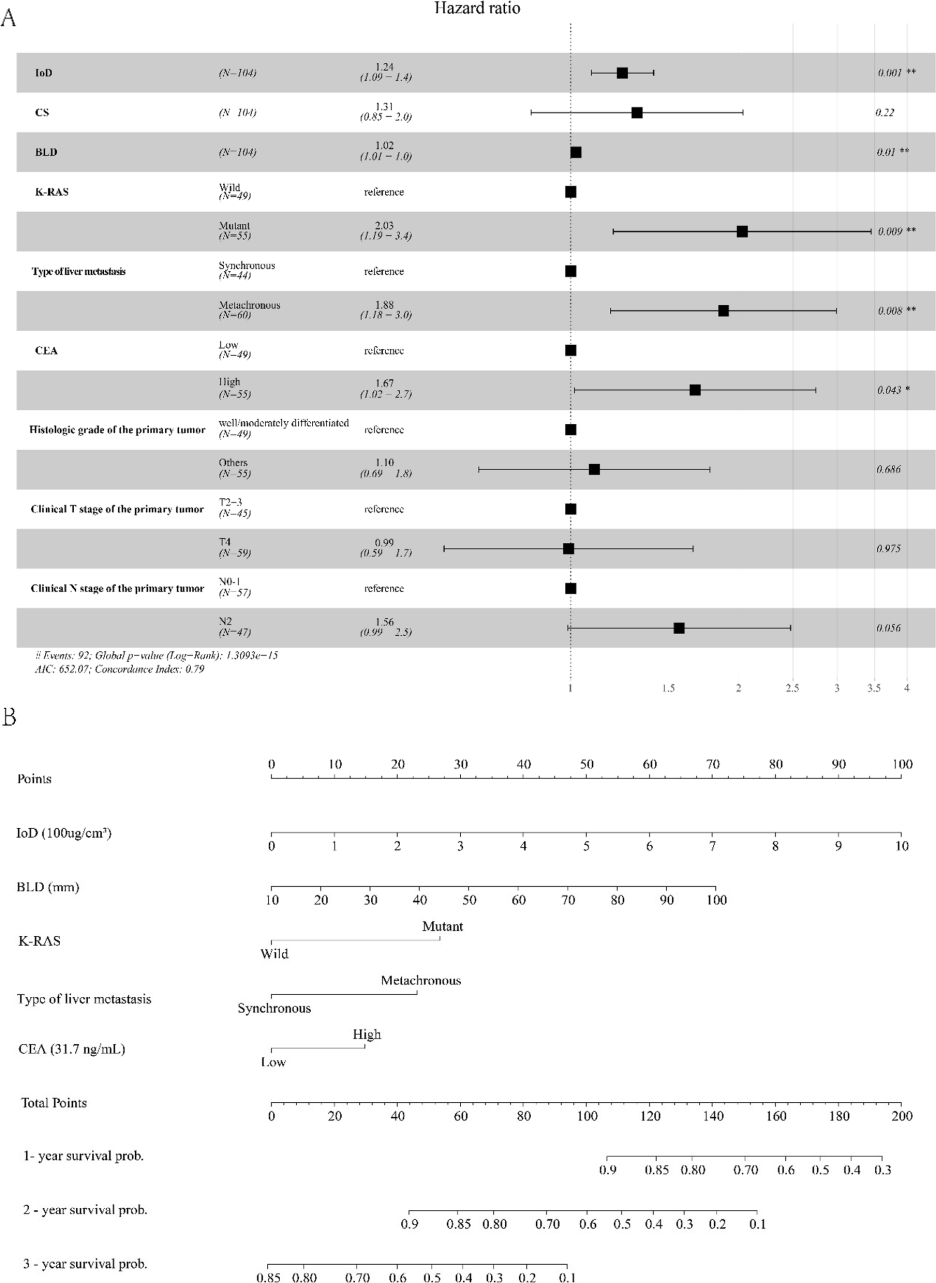
**A**. Five variables were screened out for constructing a nomogram. **B**. The nomogram was constructed based on Cox proportional hazards regression model, including IoD, BLD, K-RAS, Type of liver metastasis, and CEA

NIOD was excluded from the multivariate Cox analysis (VIF = 13.328). Multivariate Cox analysis showed that the IoD (hazard ratio [HR]: 1.238; 95% confidence interval [95% CI]: 1.089–1.408; *P* < 0.001), BLD (HR: 1.022; 95% CI: 1.005–1.038, *P* = 0.010), higher baseline CEA (HR: 1.670; 95% CI: 1.016–2.745, *P* = 0.043), K-RAS mutation (HR: 2.027; 95% CI: 1.192–3.449; *P* = 0.009), and metachronous liver metastasis (HR: 1.877; 95% CI: 1.179–2.988; *P* = 0.008) were independent risk factors for OS in patients with CRLM, Table 2, Fig. 3.

**Table 2:** Univariate and multivariate associations of baseline data and spectral CT parameters with Overall survival

Variables	Univariate analysis		Multivariate analysis	
	HR (95% CI)	*p* Value	HR (95% CI)	*p* Value
IoD	1.383 (1.268–1.508)	** < 0.001**	1.238(1.089–1.408)	** < 0.001**
CS	2.347 (1.845–2.985)	** < 0.001**	1.314 (0.849–2.034)	0.219
CEA	2.284 (1.483–3.517)	** < 0.001**	1.670 (1.016–2.745)	**0.043**
≥ 31.7 ng/mL versus < 31.7 ng/mL				
BLD (mm)	1.025 (1.010–1.040)	** < 0.001**	1.022 (1.005–1.038)	**0.010**
Boundary	1.112 (0.738–1.675)	0.612		
Clear versus Unclear				
Texture	0.866 (0.573–1.310)	0.496		
Homogeneous versus Heterogeneous				
Shape	0.749 (0.493–1.141)	0.179		
Round versus lobulated				
Liver oligometastasis	0.991 (0.636–1.547)	0.971		
No and Yes				
Histologic grade of the primary tumor	0.630 (0.415–0.956)	**0.029**	1.103 (0.686–1.775)	0.686
Well/moderately differentiated versus Others				
K-RAS	3.057 (1.954–4.781)	** < 0.001**	2.027 (1.192–3.449)	**0.009**
Mutant versus Wild type				
Location of the primary tumor	1.305 (0.867–1.964)	0.203		
Colon versus Rectal				
Clinical T stage of the primary tumor	2.158 (1.396–3.336)	** < 0.001**	0.992 (0.594–1.656)	0.975
T4 versus T2/T3				
Clinical N stage of the primary tumor	1.945 (1.277–2.962)	**0.002**	1.565 (0.989–2.474)	0.056
N2 versus N1-0				
Type of liver metastasis	1.857 (1.218–2.832)	**0.004**	1.877 (1.179–2.988)	**0.008**
Metachronous versus Synchronous				
Sites of metastases	1.150 (0.694–1.908)	0.587		
Liver and extrahepatic versus Liver only				
Systemic therapy scheme	1.167 (0.551–1.333)	0.493		
FOLFOX versus FOLFIRI				
2-RECIST1.1	1.103 (0.481–2.530)	0.817		
R + versus R-				

Note: *HR* Hazard ratio, *IoD* Iodine concentration, *CS* Spectral curve slope, *CEA* Carcinoembryonic antigen, *BLD* Baseline longest diameter

### Univariate and multivariate associations of baseline data and spectral CT parameters with R − 

Single factor analysis showed that IoD, CS, CEA, K-RAS mutation, and clinical T stage and N stage of the primary tumor were associated with R − , Table 3. Multivariate logistic analysis showed that IoD (OR: 2.243; 95% CI: 1.405–4.098; *P* = 0.002) and clinical N stage of the primary tumor (OR: 4.998; 95% CI: 1.210–25.345; *P* = 0.035) was an independent predictor of R − in patients with CRLM, Table 4.

**Table 3:** Univariate associations of baseline data and spectral CT parameters with responders

Variables	Overall (*N* = 104)	R + (*N* = 28)	R- (*N* = 76)	*t/Z/*χ*2*	*p*
IOD	5.82 (3.11,7.87)	2.24 (1.76,3.80)	7.11 (5.22,8.76)	-6.490	** < 0.001**
CS	1.17 (0.55,1.95)	0.37 (0.29,0.80)	1.77 (1.01,2.04)	-5.896	** < 0.001**
Year	57.73 ± 12.56	56.68 ± 10.44	58.12 ± 13.30	-0.517	0.607
BLD (mm)	25.74 (20.55,30.18)	23.37 (18.83,28.41)	26.08 (21.00,31.54)	-1.422	0.155
CEA	32.91 (17.00,96.40)	21.02 (5.25,46.47)	38.84 (19.50,145.00)	-2.748	**0.006**
Sex				2.962	0.085
Female	44 (42.3%)	8 (28.6%)	36 (47.4%)		
Male	60 (57.7%)	20 (71.4%)	40 (52.6%)		
Primary tumor				0.586	0.444
Rectum	53 (51.0%)	16 (57.1%)	37 (48.7%)		
Colon	51 (49.0%)	12 (42.9%)	39 (51.3%)		
Sites of metastases				—	0.423*
Liver only	83 (79.8%)	24 (85.7%)	59 (77.6%)		
Liver and extrahepatic	21 (20.2%)	4 (14.3%)	17 (22.4%)		
Liver oligometastasis				0.801	0.371
NO	71 (68.3%)	21 (75.0%)	50 (65.8%)		
YES	33 (31.7%)	7 (25.0%)	26 (34.2%0		
Type of liver metastasis				3.455	0.063
Synchronous	44 (42.3%)	16 (57.1%)	28 (36.8%)		
Metachronous	60 (57.7%)	12 (42.9%)	48 (63.2%)		
Clinical T stage of the primary tumor				6.894	**0.009**
T2-3	45 (43.3%)	18 (64.3%)	27 (35.5%)		
T4	59 (56.7%)	10 (35.7%)	49 (64.5%)		
Clinical N stage of the primary tumor				—	0.001*
N0-1	57 (54.8%)	23 (82.1%)	34 (44.7%)		
N2	47 (45.2%)	5 (17.9%)	42 (55.3%)		
Histologic grade of the primary tumor				0.943	0.332
Well/moderately differentiated	49 (47.12%)	11 (39.3%)	38 (50.0%)		
Others	55 (52.88%)	17 (60.7%)	38 (50.0%)		
K-RAS				6.616	**0.010**
Wild	49 (47.1%)	19 (67.9%)	30 (39.5%)		
Mutant	55 (52.9%)	9 (32.1%)	46 (60.5%)		
Boundary				0.057	0.812
Clear	54 (51.9%)	14 (50.0%)	40 (52.6%)		
Unclear	50 (48.1%)	14 (50.0%)	36 (47.4%)		
Texture				0.042	0.838
Homogeneous	54 (51.9%)	15 (53.6%)	39 (51.3%)		
Heterogeneous	50 (48.1%)	13 (46.4%)	37 (48.7%)		
Shape				3.212	0.073
Round	63 (60.6%)	13 (46.4%)	50 (65.8%)		
Lobulated	41 (39.4%)	15 (53.6%)	26 (34.2%)		
Systemic therapy scheme				2.628	0.105
FOLFIRI and bevacizumab	72 (69.2%)	16 (57.1%)	56 (73.7%)		
FOLFOX and bevacizumab	32 (30.8%)	12 (42.9%)	20 (26.3%)		

Note: * Fisher exact probability test

**Table 4:** Multivariate associations of baseline data and spectral CT parameters with responder

Variables	Multivariate analysis			
	Coefficients	*Z* value	OR (95% CI)	*p* Value
IoD	0.808	3.117	2.243 (1.405–4.098)	**0.002**
CS	0.406	0.553	1.501 (0.315–6.766)	0.580
CEA	0.001	0.341	1.001 (0.997–1.006)	0.733
K-RAS	-0.631	-0.862	0.532 (0.116–2.136)	0.389
Wild type versus mutant				
Clinical T stage of the primary tumor	0.227	0.323	1.255 (0.306–5.007)	0.747
T4 versus T2/T3				
Clinical N stage of the primary tumor	1.609	2.108	4.998 (1.210–25.345)	**0.035**
N2 stage versus N0-1				

**Fig. 4:**
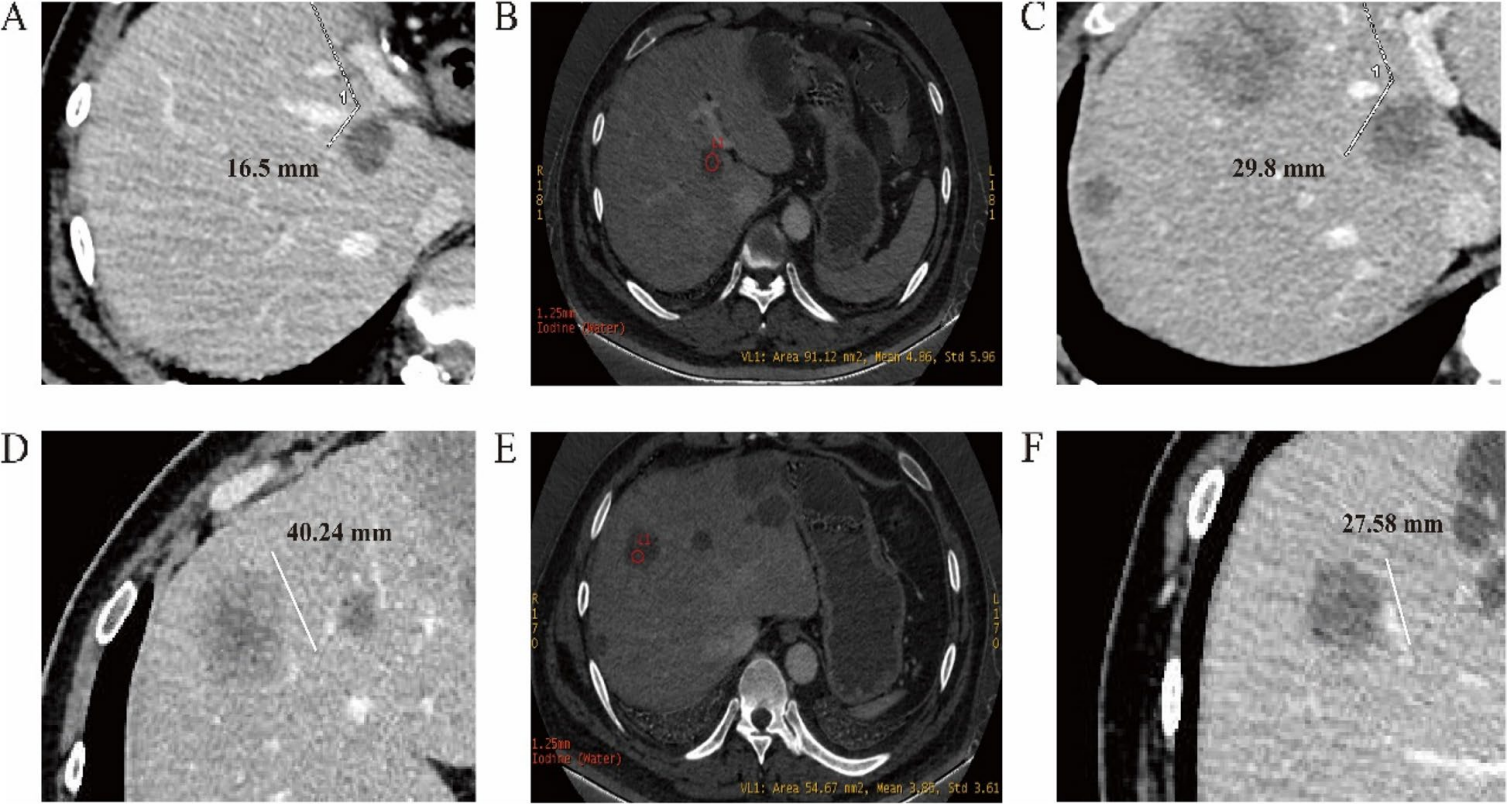
Baseline contrast-enhanced CT, spectral CT within 2 months, and contrast-enhanced CT at 6-month in R − and R + patients. A-C. For R − patients, the mean value of IoD was 4.86 (100ug/cm^3^). D-F. For R + patients, the mean value of IoD was 3.85 (100ug/cm^3^)

**Fig. 5:**
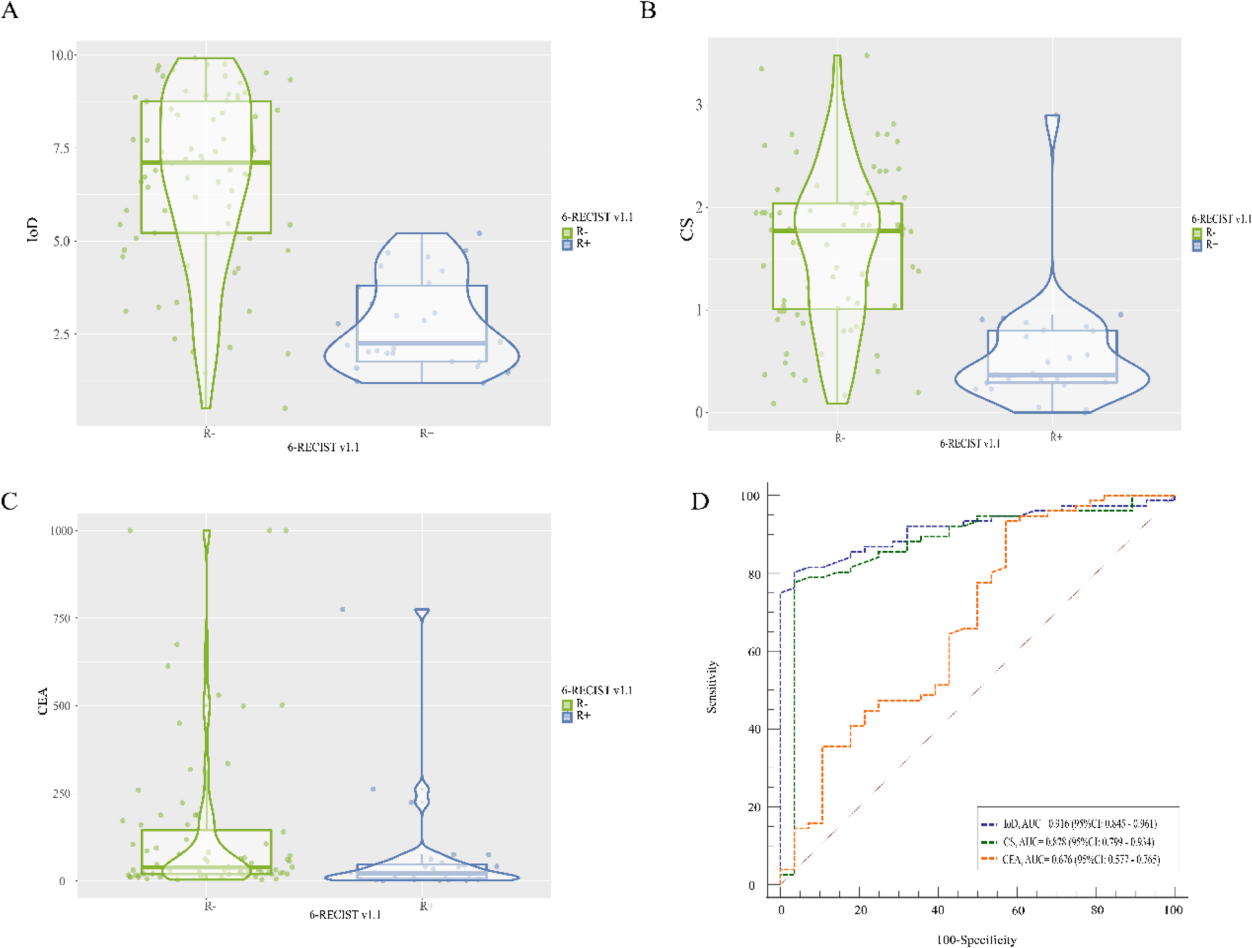
A-C. IoD, CS, and baseline CEA differences between the R + and R − groups at 6-RECIST v1.1. D. AUC of IoD, CS, and baseline CEA in R + and R − groups

All 104 CRLM patients were divided into R + (*n* = 28) and R − (*n* = 76) groups. Comparison of spectral CT parameters revealed significant differences between the two groups in IOD, CS, and baseline CEA. The R + group had lower IoD, CS and baseline CEA early during the treatment process, Figs. 4, and 5. Using IoD cutoff values of 4.75 (100ug/cm^3^), the AUC was 0.916, sensitivity and specificity were 80.3% and 96.4%, positive and negative predictive value were 0.984 and 0.643, respectively, Fig. 5 D.

## Discussion

Early evaluation of the efficacy of first-line chemotherapy combined with bevacizumab in patients with CRLM is critical for improving patient outcomes. The 2-month portal venous phase spectral CT analysis for early quantitative assessment of the efficacy of FOLFOXIRI with bevacizumab in patients with CRLM showed that IoD was an independent predictor of R + . IoD, baseline CEA, BLD, K-RAS mutation, and metachronous liver metastasis were independent risk factors for OS after treatment for CRLM. However, based on 2-RECIST v1.1, there were no significant associations with OS in patients with CRLM. In addition, IoD had good discriminative performance for R − defined by 6-RECIST v1.1, with AUC of 0.916, sensitivity and specificity were 80.3% and 96.4%, positive and negative predictive value were 0.984 and 0.643, respectively. These observations indicated that IoD on portal venous phase spectral CT can reflect treatment efficacy in patients with CRLM administered FOLFOXIRI combined with bevacizumab within 2 months of commencing treatment.

Previous studies [24, 25] showed that the K-RAS mutations are negatively associated with OS in CRLM patients. Our results also found that K-RAS mutation was an independent predictor of OS after first-line chemotherapy combined with bevacizumab in patients with CRLM. The reason could be the difference in response to systemic chemotherapy according to the K-RAS mutation status. In the context of systemic chemotherapy, *Zimmitti G *et al. [26] investigated the association between RAS mutational status and response to preoperative chemotherapy in patients with CRLM. They revealed that RAS mutations were significantly associated with minor pathological and suboptimal morphological responses. Other studies also demonstrated that K-RAS mutations were significantly associated with minor response to chemotherapy in patients with CRLM, and that RAS mutation status may serve as a biomarker for response to chemotherapy [27, 28]. In addition, higher baseline CEA (≥ 31.7 ng/mL), BLD, and metachronous liver metastasis were independent risk factors for OS after FOLFOXIRI combined with bevacizumab for CRLM. We believe that the size of liver metastases at baseline affects the penetration of chemotherapy drugs into the tumor. Previous studies have also shown that the size of liver metastases at baseline and the size of metastases at the first follow-up are important prognostic predictors of CRLM [29, 30]. Therefore, systemic therapy should be combined with transcatheter arterial chemoembolization or radiofrequency ablation to improve the long-term survival of patients with large baseline (≥ 25.77 mm) liver metastases [30]. Furthermore, univariate analysis showed that the T stage and N stage of the primary tumor, and histologic grade of the primary tumor were associated with the OS of CRLM, in line with several previous studies in CRLM [31, 32].

Compared with traditional mixed-energy CT, spectral CT has diverse imaging parameters, including base material and single-energy CT imaging. Previous studies showed that the IoD can reflect the tumor microvessel density [22, 33]. Tumor microvessels lack the normal vascular wall composition, resulting in retention or extravasation of iodine in the tumor. *Drljevic-Nielsen *et al*.* [34] used the dual-energy CT IoD for early prediction of the efficacy of targeted therapy for metastatic renal cell carcinoma. IoD at baseline and after 1 month of chemotherapy were independent risk factors for outcomes in patients with mRCC. In addition, *Luo *et al*.* [22] also reported that the NIoD can predict early recurrence of hepatocellular carcinoma after surgery. Histopathological analysis showed that the NIoD was positively correlated with microvessel density in hepatocellular carcinoma. Therefore, the difference in the NIoD reflects treatment efficacy. In the present study, IoD within 2 months of treatment were also independent risk factors for OS in patients with CRLM. In addition, there was an association between lower CS and OS with CRLM in univariate analysis. We believe that the higher IOD [≥ 4.75 (100ug/cm^3^)] in CRLM after FOLFOXIRI combined with bevacizumab indicates that the microvascular structure of the tumor is not effectively inhibited or destroyed. Therefore, IoD was found to be an independent predictor of CRLM by multivariate logistic regression. However, some of the previous studies have suggested that baseline and post-treatment CT parameters do not predict survival outcomes in patients with CRLM [35, 36]. Our findings suggest that spectral CT parameters may be a useful biomarker for noninvasively predicting early efficacy response and survival outcomes in patients with CRLM.

This study had some limitations. First, as this was a retrospective study with a small sample size from a single center, the dynamic changes in multiparametric spectral CT parameters before and after chemotherapy for CRLM could not be determined. Second, there may have been differences in the treatment options among patients, but FOLFIRI or FOLFOX with bevacizumab is recommended as a first-line chemotherapy regimen for CRLM in previous studies [37]. Third, as only a small number of patients in our cohort underwent surgical resection of liver metastasis after chemotherapy, histopathological analysis of liver metastasis was not included in this study. However, this appears to be important based on previous studies of the HGP of liver metastasis [38]. Finally, we used spectral CT only in the portal venous phase and acquired spectral CT parameters for only a single target lesion. Further prospective studies are required including baseline spectral CT in patients with CRLM to further validate the correlations of the spectral CT parameters of multiple target lesions with survival outcomes and early response.

## Conclusions

In conclusion, spectral CT IoD can predict the OS and responder of patients with CRLM after 2 months of treatment with bevacizumab-containing therapy. At the same time, baseline CEA, BLD, K-RAS mutation, and metachronous liver metastasis were independent risk factors for OS after treatment for CRLM.

## Data Availability

The datasets used and/or analysed during the current study are available from the.corresponding author on reasonable request.

## Abbreviations

CRLM: Colorectal cancer liver metastasis
IoD: Iodine concentration
NIoD: Normalized iodine concentration
OS: Overall survival
RECIST v1.1: Response Evaluation Criteria in Solid Tumors Version 1.1
CR: Complete response
PR: Partial response
SD: Stable disease
PD: Progressive disease
R +: Responders
R −: Non-responders
